# Proteome Dynamics of Persulfidation in Leaf Tissue under Light/Dark Conditions and Carbon Deprivation

**DOI:** 10.3390/antiox12040789

**Published:** 2023-03-23

**Authors:** Ana Jurado-Flores, Cecilia Gotor, Luis C. Romero

**Affiliations:** Instituto de Bioquímica Vegetal y Fotosíntesis, Consejo Superior de Investigaciones Científicas and Universidad de Sevilla, Avenida Américo Vespucio, 49, 41092 Sevilla, Spain

**Keywords:** carbon starvation, light photoperiod, persulfidation, quantitative proteomics, sulfur assimilation

## Abstract

Hydrogen sulfide (H_2_S) acts as a signaling molecule in plants, bacteria, and mammals, regulating various physiological and pathological processes. The molecular mechanism by which hydrogen sulfide exerts its action involves the posttranslational modification of cysteine residues to form a persulfidated thiol motif. This research aimed to study the regulation of protein persulfidation. We used a label-free quantitative approach to measure the protein persulfidation profile in leaves under different growth conditions such as light regimen and carbon deprivation. The proteomic analysis identified a total of 4599 differentially persulfidated proteins, of which 1115 were differentially persulfidated between light and dark conditions. The 544 proteins that were more persulfidated in the dark were analyzed, and showed significant enrichment in functions and pathways related to protein folding and processing in the endoplasmic reticulum. Under light conditions, the persulfidation profile changed, and the number of differentially persulfidated proteins increased up to 913, with the proteasome and ubiquitin-dependent and ubiquitin-independent catabolic processes being the most-affected biological processes. Under carbon starvation conditions, a cluster of 1405 proteins was affected by a reduction in their persulfidation, being involved in metabolic processes that provide primary metabolites to essential energy pathways and including enzymes involved in sulfur assimilation and sulfide production.

## 1. Introduction

The gasotransmitter hydrogen sulfide (generically refers to H_2_S and the anionic forms hydrosulfide, HS^−^, and sulfide, S^2−^) acts as a signaling molecule that regulates numerous physiological and pathological processes in plants [1,2,3], bacteria [4,5], and humans and mammals in general [6,7,8]. The number of regulated processes in plants has grown very rapidly, and H_2_S has been shown to control plant physiological processes related to stress resistance [9,10], stomatal aperture and drought [11,12,13], autophagy [14,15,16], and developmental processes such as seed germination and root development [17,18,19].

Hydrogen sulfide is produced enzymatically in eukaryotic cells, but the enzymes responsible for its synthesis vary depending on the organism. Thus, in mammalian cells, the proteins cystathionine β-synthase (CBS), cystathionine γ-lyase (CSE), and 3-mercaptopyruvate sulfurtransferase (3MST) are the main enzymes responsible for its production [20]. However, in plant cells, H_2_S is synthesized by sulfite reductase as the final product of photosynthesis-dependent sulfate reduction in chloroplasts prior to its incorporation into cysteine [21]. H_2_S can be also produced by the action of D/L cysteine desulfhydrases (LCD/DCD/DES1), which breakdown cysteine in the cytosol and mitochondria [22,23,24], by ß-cyanoalanine synthase (CAS), which detoxifies cyanide in mitochondria, releasing sulfide and cyanoalanine [25,26], and by cystathionine β-synthase, as recently reported [27]. H_2_S homeostasis is maintained in plant cells by *O*-acetylserine(thiol)lyase enzymes that incorporate sulfide into *O*-acetylserine molecules to produce cysteine within the cytosol, mitochondria, and chloroplast compartments [21,28].

The persulfidation of cysteine residues is the best-studied molecular mechanism by which H_2_S exerts its signaling function, and refers to a protein posttranslational modification where a thiol (P-SH) residue of cysteine is transformed into a persulfide (P-SSH) group [29]. This modification has been widely established in mammalian cells and initially described in leaf protein extracts from mature Arabidopsis wild-type plants by using the modified biotin switch method [30], later extended and improved by using the chemoselective tag-switch method to detect persulfidated proteins in the leaves of wild-type and *des1* mutant lines [31]. Since then, the number of proteins that can be modified by persulfidation has grown substantially [14,32]. Protein persulfidation in plants results in changes in the catalytic capacity of enzymes [30] or their subcellular location [33], regulating important physiological processes such as autophagy through the modification of the autophagy-related protein 4 (ATG4) and ATG18 [14,15], stomatal opening via DES1, Respiratory burst oxidase protein D (RBOHD) and SNF Related kinase 2 (SnRK2) enzyme activation in guard cells [12,34], hormone signaling [35], and seed germination [17] through Abscisic acid insentive 4 (ABI4) protein modification.

Currently, almost 13% of the Arabidopsis proteome is persulfidated under normal growth conditions, but the persulfidation profile is significantly altered by nutritional changes in the plant or upon abscisic acid-induced autophagy [14,32]. Although protein persulfidation has emerged as the main mechanism of action of H_2_S signaling processes, the sulfide molecule is the lowest redox state (−2) of the sulfur atom and cannot react directly with reduced cysteine thiol, requiring the presence of an oxidant. Based on thermodynamic and kinetic aspects, the more plausible explanation for H_2_S-mediated persulfidation is the reaction with oxidized cysteine residues as sulfenic acid since the reaction with disulfide occurs at a very slow rate [36,37,38]. It has been proposed that H_2_S production and protein persulfidation are used by the cell to resolve sulfenylation and preserve protein function, preventing the irreversible oxidation of cysteine residues [39,40]. With these considerations, it is evident that there should be an interconnection between the reactive oxygen species (ROS) production–physiological processes, sulfide production, and the formation of protein persulfides. 

Oxidant species are produced in plant cells in the chloroplasts during the light reaction of photosynthesis, the mitochondrial respiratory electron transport chain, and the action of multiple organellar and plasma membrane oxidases. Many low molecular reductants and antioxidant molecules are synthesized in the plant cell, such as carotenoids and flavonoids, with ascorbate and glutathione being key components that regulate the overall redox state [41,42]. The production of ROS or a decrease in antioxidant capacity is used as a redox signaling process to link the environmental or developmental status with the cellular metabolism [43]. Different signaling and metabolic processes are regulated through the thiol redox state of protein cysteine residues, which regulates protein structure and function [44]. This is the case for electrons produced during photosynthesis that provide energy for carbon reduction but also regulate metabolic pathways through the reduction of specific oxidized protein cysteine residues via thioredoxins. Several proteomic approaches have been used to unveil the dynamics of cellular redox changes and the link with cellular metabolism by observing the thiol redox states of proteins and their functional implications [45,46,47]. These proteomic approaches focus on the study of the reduced or oxidized thiol states mediated by ROS or RNS and only consider the disulfide bond (S-S), *S*-sulfenylation (-SOH), *S*-nitrosylation (-SNO), and *S*-glutathionylation (-SSG) as reversible oxidized thiol states, overlooking the well-established thiol persulfide (-SSH) modification. Under conditions of high and persistent oxidative stress, oxidized thiols can progress to an overoxidized state as sulfinic (-SO_2_H) and sulfonic acid (-SO_3_H) motifs that are irreversible [48]. Protein persulfidation can be considered as a protective mechanism against protein oxidation since the overoxidized motifs of persulfide, such as perthiosulfenic (-SSOH), perthiosulfinic (-SSO_2_H), or perthiosulfonic (-SSO_3_H), can be reduced back to thiol by GSH or thioredoxins [39,40]. 

In this work, we wanted to delve into the dynamic changes in the protein persulfidation profile of the leaf proteome to identify cellular processes that interconnect the signaling/protective function of sulfide with important environmental processes that impact the metabolism and physiology of the plant. With this objective, we have studied the changes in the protein persulfidation profiles in two processes with an important impact on the physiology of the plant, such as the light changes during the day photoperiod, regulating the photosynthetic activity of the plant, and on the other hand, the nutritional carbon deficiency, closely linked to C fixation during photosynthesis.

## 2. Materials and Methods

### 2.1. Plant Material, Treatments, and Protein Extraction

*Arabidopsis thaliana* wild-type plants were grown in soil for 30 days with a long-day light regime of 16 h of light (120 µE m^−2^ s^−1^) at 22 °C and 8 h of dark at 20 °C, as described in [49]. For exposure to carbon deprivation, 30-day-old plants were transferred to continuous darkness for 3 d (sample set 1) and then transferred back to the light photoperiod to recover for an additional 5 d (sample set 3) [50]. For proteomic analysis, control plants grown for 38 days with a long-day light photoperiod were used (sample sets 2 and 4). *Arabidopsis thaliana* seeds were provided by The Nottingham Arabidopsis Stock Centre (NASC, University of Nottingham, Nottingham, UK).

### 2.2. Quantification of Hydrogen Sulfide Using Liquid Chromatography with Tandem Mass Spectrometry (LC-MS/MS)

Hydrogen sulfide was quantified following a previously described method [51]. Approximately 150 mg of frozen leaf tissue was homogenized in 1.5 mL Eppendorf tubes twice for 2 min at maximum speed within a Retsch ball mill MM400 (Retsch GmbH, Haan, Germany). The metabolites were extracted from each aliquot in 300 µL of a homogenous mixture of Tris-HCl buffer (100 mM, pH 8.5; Ethylenediaminetetraacetic acid (EDTA) 1 mM), with shaking for 30 min at 4 °C. Samples were centrifuged for 15 min at 21,130× *g* at 4 °C. 

A 25 μL sample extract was mixed with 30 μL of MBB (monobromobimane) solution (20 mM). The mixture was vortexed, incubated for 60 min at room temperature, and the derivatization reaction stopped by adding 5 μL of 20% formic acid. The mixture was subjected to centrifugation at 21,130× *g* for 10 min. 

The derivative sulfide dibimane was analyzed through LC-MS/MS using the ExionLC™ UPLC system (Sciex, Framingham, MA, USA) with a reversed-phase column (100 mm × 4.6 mm × 100 Å particle, Kinetex XB-C18) (Phenomenex, Torrance, CA, USA). Chromatography and mass spectrometry analyses were performed as previously described [52].

### 2.3. Immunoblot Analysis

Plant leaf material (200 mg) was ground in liquid nitrogen with 400 μL of extraction buffer (50 mM Tris-HCl, pH 7.5, NaCl 100 mM, 1 mM EDTA, 1 mM phenylmethylsulfonyl fluoride, and 4% (*v*/*v*) protease inhibitor cocktail (Roche, Basel, Switzerland)) using a mortar and pestle and centrifuged at 6000× *g* for 5 min to obtain the supernatant fraction. The centrifugation was repeated three times until the supernatant was free of turbidity. The total amount of protein in the resulting supernatant was determined using a previously described method [53]. For immunoblot analyses, 40 μg of leaf protein extracts was separated using sodium dodecyl sulfate polyacrylamide gel electrophoresis (SDS-PAGE) through a 10% (*w*/*v*) polyacrylamide gel before being transferred to a nitrocellulose membrane (Bio-Rad Laboratories, Inc., Hercules, CA, USA) according to the manufacturer’s instructions. Custom-made polyclonal anti-recombinant OAS-A1 antibody (Biomedal S.L., Sevilla, Spain) and secondary antibodies were diluted 1:10000 in Phosphate buffered saline (PBS) solution containing 0.1% (*v*/*v*) Tween 20 (Sigma-Aldrich, Merck KGaA, Darmstadt, Germany) and 5% (*w*/*v*) milk powder. The ECL Select Western Blotting Detection Reagent (GE Healthcare Technology, Chicago, USA) was used to detect proteins with horseradish peroxidase-conjugated anti-rabbit secondary antibodies. For a protein loading control, the membrane before immunodetection was stained with Ponceau S (Sigma-Aldrich, Merck KGaA, Darmstadt, Germany) to detect all protein bands.

### 2.4. Persulfidation Protein Enrichment and Quantitation Using Label-Free Analysis

Protein samples from four biological replicates of leaf tissues were isolated from 1 g of ground leaf material in a mortar under liquid nitrogen. Enriched cytosolic extract was obtained from ground material homogenized in a solution containing 0.125 parts (*v*/*w*) of buffer I [50 mM Tris, pH 7.1, amended with 100 mM KCl, 20% glycerol and protease inhibitor cocktail (25×) (Roche, Basel, Switzerland)] and 0.05 parts (*v*/*w*) of buffer II [1 mM pepstatin and 1.4 μM phenymethysulfonyl fluoride dissolved in ethanol] and then centrifuged at 257,300× *g* for 1 h at 4 °C. One milligram of protein per sample was submitted to tag-switch labeling for persulfidation protein enrichment as described in [31]. After elution of the labeled proteins from the streptavidin beads, they were precipitated through the trichloroacetic acid/acetone procedure. The protein pellet was solubilized at 56 °C for 10 min in digestion buffer containing 100 mM Tris-HCl, pH 8.5, with 2% (*w*/*v*) sodium deoxycholate, 5 mM tris(2-carboxyethyl)phosphine, and 30 mM 2-chloroacetamide for reduction and protein alkylation. Trypsin and Lys-C were added at ratios of 1:50 and 1:100 (*w*/*w*), respectively, to the protein samples and digested overnight at 37 °C with shaking. Sodium deoxycholate was removed through precipitation with trifluoroacetic acid, which was added until the pH reached 2, and centrifuged at 18,407× *g* for 10 min. The supernatant containing the digested peptides was desalted using an Oasis HLB plate (Waters Corporation, Milford, MA, USA), dried, and stored at −80 °C.

A total of 1000 ng of the tryptic peptide mixture dissolved in 2% formic acid was analyzed using an Ultimate3000 high-performance liquid chromatography system (Thermo Fisher Scientific Inc., Waltham, MA, USA) coupled on-line to a Q Exactive HF-x mass spectrometer (Thermo Fisher Scientific Inc., Waltham, MA, USA). Buffer A consisted of water acidified with 0.1% formic acid, while Buffer B was 80% acetonitrile and 20% water with 0.1% formic acid. The peptides were first trapped for 1 min at 30 µL/min with 100% buffer A on a trap (0.3 × 5 mm with PepMap C18, 5 μm-100Å Thermo Fisher Scientific Inc., Waltham, MA, USA); after the trapping, peptides were separated using a 50 cm analytical column packed with C18 beads (Poroshell 120 EC-C18, 2.7 μm, Agilent, Santa Clara, CA, USA). The gradient was 9–40%B in 155 min at 400 nL/min. Buffer B was then raised to 55% in 10 min and increased to 99% for the cleaning step. The peptides were ionized using a spray voltage of 1.9 kV and a capillary heated to 275 °C. The mass spectrometer was set to acquire full-scan MS spectra (350–1400 *m*/*z*) for a maximum injection time of 120 ms at a mass resolution of 120,000 and an automated gain control (AGC) target value of 3 × 106. Up to 25 of the most intense precursor ions were selected for tandem mass spectrometry (MS/MS). HCD fragmentation was performed in the HCD cell, with the readout in an Orbitrap mass analyzer at a resolution of 15,000 (isolation window of 1.4 Th) and an AGC target value of 1 × 105 with a maximum injection time of 25 ms and a normalized collision energy of 27%.

### 2.5. Proteomic Data Analysis

All raw files were analyzed in MaxQuant v1.6.17 software (Max Planck Institute of Biochemistry, Planegg, Germany) using the integrated Andromeda Search engine, and were searched against the *Arabidopsis thaliana* UniProt Reference Proteome without isoforms (July 2020 release with 39,284 protein sequences). MaxQuant was used with the standard parameters (the “Label-Free Quantification (LFQ)” and “Match between runs” were selected with automatic values) except for the following modifications: carbamidomethyl (C) was set as a variable modification together with oxidation (M), acetylation (Protein N-term), and deamidation (N) [54]. Data analysis was carried out with Perseus v1.6.14 (Max Planck Institute of Biochemistry, Germany), and the LFQ intensities found in ‘proteingroups.txt’ were filtered for reverse and potential contaminants [55]. For quantitation, the proteins found in at least three biological replicates were used, and missing values were input with the automatic settings and further statistical analysis as described in [56]

The mass spectrometry proteomics data have been deposited in the ProteomeXchange Consortium [57] via the PRIDE partner repository (EMBL-EBI, Hinxton, UK) with the dataset identifier PXD024061.

Classification of the proteins by their assignment and Gene Ontology in biological processes were performed and analyzed using UniProt (EMBL-EBI, Hinxton, UK) and MapMan (Max Planck Institute for Molecular Plant Physiology, Germany) nomenclature developed for plant-specific pathways and processes [58]. Kyoto Encyclopedia of Genes and Genomes (KEGG) pathway enrichment of the proteins within clusters was performed with the Database for Annotation, Visualization and Integrated Discovery (DAVID) (LHRI/ADRD at Frederick National Laboratory, Frederick, MD, USA) web platform [59].

### 2.6. Protein Persulfidation In-Gel Detection 

Protein persulfidation patterns in plant tissue were analyzed with the dimedone switch method as previously described [60]. In detail, plant leaf material (100 mg) was ground in liquid nitrogen with 200 μL of cold PBS lysis buffer (1X PBS pH 7.4, 1 mM EDTA, 2% (*w*/*v*) SDS) supplemented with 1X protease inhibitor (Pierce™, Thermo Scientific). Then, samples were incubated with 5 mM 4-chloro-7-nitrobenzofurazan (Cl-NBF) at 37 °C for 30 min, protected from light. Methanol/chloroform precipitation was performed to eliminate excess Cl-NBF, and the protein pellets obtained were washed with cold methanol, dried, and redissolved in 1X PBS with 2% (*w*/*v*) SDS supplemented with 1X protease inhibitor (Pierce™, Thermo Fisher Scientific Inc., Waltham, MA, USA). Proteins were then incubated with 25 µM DAz-2:Cy-5 preclick mix at 37 °C for 30 min. Following incubation, methanol/chloroform precipitation was performed, and the pellets were washed with methanol as described above. Protein labeling was analyzed using SDS-PAGE. After SDS-PAGE, gels were fixed for 30 min in 12.5% methanol and 4% acetic acid protected from light. The gel was imaged at 640 nm for the Cy5 signal and 488 nm for the NBF-Cl signal on a Typhoon FLA 9500 (GE Healthcare Technology, Chicago, IL, USA). The persulfidation levels were quantified by measuring the Cy5/Cl-NBF fluorescence signal ratio.

## 3. Results

### 3.1. Light/Dark Conditions Regulate the Basal Protein Persulfidation Level in Leaf Tissues

Previous proteomic analyses in leaf and root tissues have shown that a significant number of proteins contain cysteine residues modified by persulfidation of the thiol residues (R-SSH), which can represent up to 13% of the total Arabidopsis proteome [31,32]. To further elucidate the regulation of protein persulfidation mediated by H_2_S, we used a label-free quantitative (LFQ) approach combined with the tag-switch method to measure the protein profile of persulfidation in leaf samples under different growth physiological conditions, such as light regimen and carbon nutrient deprivation, which have an important impact on cell metabolism.

**Figure 1:**
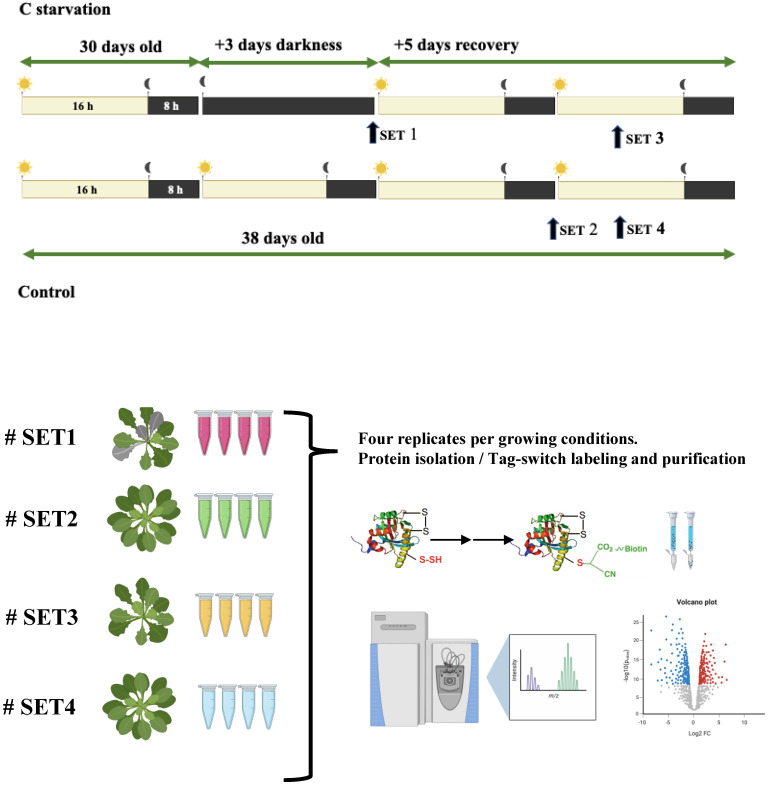
Proteomic analysis of leaf protein persulfidation in response to light conditions and carbon starvation. Workflow of leaf sample preparation followed by tag-switch protein labeling, persulfidated protein purification, tryptic digestion, and quantitative Data-independent acquisition (DIA) analysis of eluted proteins.

Carbon fixation is achieved through photosynthesis and, therefore, linked to the light conditions. Carbon nutrient starvation was attained in leaves of 30-day-old mature plants under a long-day photoperiod subjected to continuous darkness for three days followed by recovery for 5 additional days. Leaf samples were collected at two different stages of induced carbon starvation, at the end of day three of continuous darkness (sample set 1, S1) and at the middle of the day after 5 days of recovery (sample set 3, S3) (Figure 1). For comparison, control plants grown under a long-day photoperiod were collected at two different stages, the end of the dark period at 37 days of growth (8 h darkness, sample set 2, S2) and the middle of the light period on the next day (8 h light, sample set 4, S4). Proteins from four biological replicates (independent pools) of the indicated leaf tissues were isolated and subjected to the tag-switch procedure. The isolated labeled persulfidated proteins were analyzed using a label-free quantitative (LFQ) approach (Figure 1).

The proteomic analysis identified 4318 proteins in S1 samples (Dataset S1), 4211 in S2 samples (Dataset S2), 4343 in S3 samples (Dataset S3), and 4281 in S4 samples (Dataset S4), with a False discovery rate (FDR) threshold of 1%. The discovery analysis showed a total of 4599 persulfidated proteins quantified in whole-leaf samples, of which 3147 proteins were differentially present in the four sample groups as determined using ANOVA testing (FDR < 0.05) across the four biological replicates (Dataset S5).

**Figure 2:**
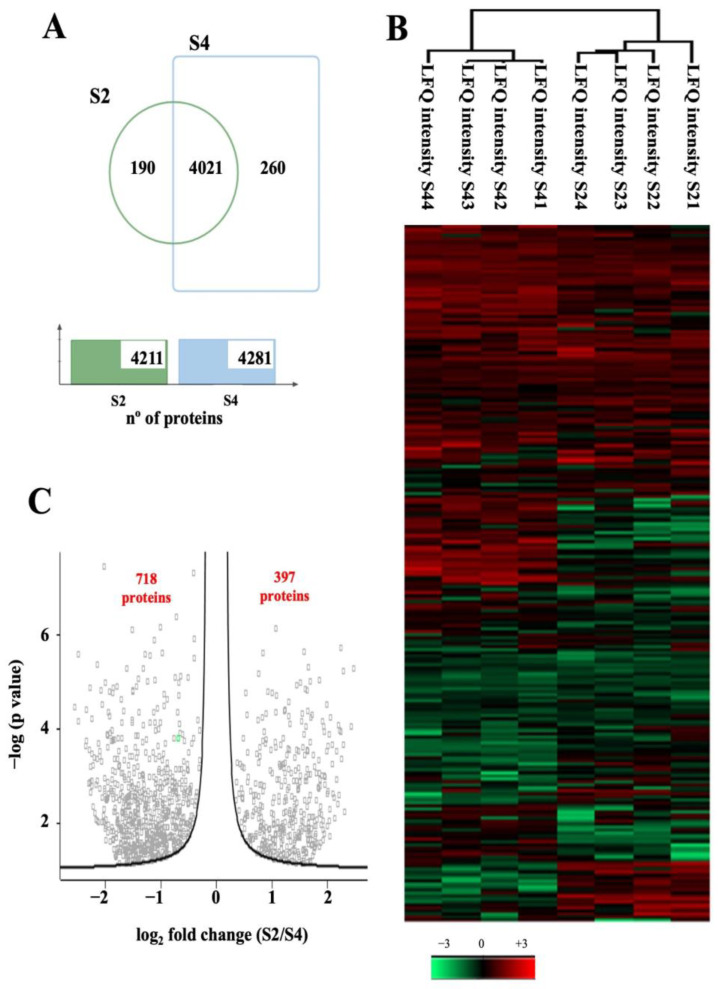
Proteomic analysis of protein persulfidation in response to light conditions. (**A**) Venn diagram showing the intersection of the persulfidated proteins identified in the sample sets collected in the dark (S2) and light (S4) photoperiod. (**B**) Hierarchical clustering of persulfidated proteins in dark-period collected samples (S2) and light-period collected samples (S4). (**C**) Volcano plot illustrating significantly differentially abundant proteins. The –log_10_ (*p* value) is plotted as the log_2_ of the fold change between dark-period collected samples (S2) and light-period collected samples (S4).

Comparison analysis of the identified proteins in the leaf samples grown under normal conditions but collected at different photoperiod stages (after 8 h of darkness, samples S2, and after 8 h of light, samples S4) showed that most proteins were common in both conditions, although 190 proteins were identified only in the dark-period collected samples and 260 in light-period samples (Figure 2A). Hierarchical clustering analysis of these plants correctly assigned replicates to the experimental groups and showed a high correlation between each of the control sample S2 and S4 replicas (Figure 2B). Quantitative analysis of the identified proteins showed that 1115 proteins were differentially persulfidated, of which 718 (i.e., 64%) showed a higher level of persulfidation in the light, and 397 (i.e., 36%) increased their levels during the dark period (Figure 2C) (Dataset S6). 

Considering both the proteins present only in one condition and those differentially more persulfidated, we can account for 544 proteins more persulfidated in the dark (S2 samples) (Dataset S7) and 913 more persulfidated in the light (S4 samples) (Dataset S8), with an assigned AGI locus code.

Sulfide-mediated protein persulfidation occurs in the presence of an oxidant or in proteins that possess oxidized thiol residues; therefore, the proteins or the processes affected by persulfidation in the light/dark period will depend on the intracellular ROS levels under those conditions. The 544 proteins more persulfidated in the dark (S2 samples) were further analyzed, and Gene Ontology (GO) by biological process and KEGG pathway enrichment and clustering were performed to classify the functions/pathways that were more affected during the dark photoperiod (Table 1). Three clusters of functions and pathways with significant enrichment scores over 2.0 were identified. The first cluster, D1, with an enrichment score of 6.6, includes GO terms related to protein folding and processing in the endoplasmic reticulum (ER). The ER provides an oxidative environment for the formation of disulfide bonds, correct protein folding, and the accumulation of many chaperone proteins. The more abundant subterm, protein folding, comprises 34 proteins that include nine molecular chaperones of the heat shock proteins 70 (HSP70) family, eight chaperonin-containing T-complex polypeptide-1 subunits (TCP-/cpn60), the calreticulins 1A, 1B, and 3, five thioredoxins, and a protein disulfide isomerase (PDIL1-2) (Table S1). The second cluster of proteins that were more persulfidated in the dark, cluster D2, comprises proteins involved in general metabolic pathways (91 proteins) and the biosynthesis of amino acids and secondary metabolites (23 and 64 proteins, respectively). Carbon metabolism in plants is finely regulated by light/dark conditions, and many of the enzymes of carbon fixation and assimilation or glycolysis show redox regulation of their activities. Among the proteins that are more persulfidated in the dark related to metabolic pathways, we identified several enzymes of the Calvin cycle, TCA cycle, and glycolysis, such as the ribulose bisphosphate-carboxylase (Rubisco) small chains (RBCS 1A, 1B, 3B), pyruvate dehydrogenase, succinate dehydrogenase (SDH1-1, SDH2-1), and several ATPase subunit proteins (Table S2). Cluster D3 includes proteins from the branched-chain amino acid and 2-oxocarboxilic acid process, such as glutamate:glyoxylate aminotransferase (GGT1) or valine-tolerant 1 (VAT-1), which have been previously described as regulated by persulfidation under nitrogen deprivation [32]. 

Under light conditions, the profile of persulfidation significantly changed with an increase in the number of modified proteins and in the processes and pathways in which they are involved, which would be expected due to the increase in reactive oxidative species generated in the light (Table 2). The main enriched cluster L1 included proteins belonging to the proteasome and the ubiquitin-dependent and ubiquitin-independent catabolic processes with 23 protein subunits of the proteolytic core 20S (Table S3). A second cluster, L2, comprises proteins of glutathione metabolism, such as glutamate-cysteine lyase (GSH1) and glutathione synthetase 2 (GSH2), which are involved in GSH biosynthesis, 10 glutathione s-transferase enzymes (GSTs), and five glutathione peroxidases (GPXs), among others (Table S3). A third enriched protein cluster, L3, includes several proteins involved in nucleosome assembly, such as the nucleosome assembly proteins NAP1;1-1;2-1;3 and four histone proteins that are more persulfidated in the light photoperiod. Glycolysis is the fourth classification term with more regulated protein persulfidation during the light photoperiod (Table S3).

### 3.2. The Persulfidation Proteome Is Reprogrammed during Carbon Deprivation

**Figure 3:**
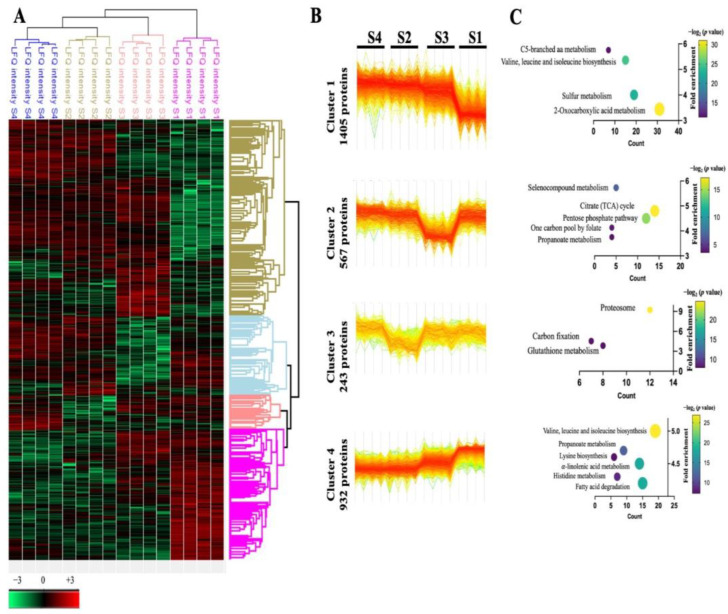
Hierarchical heatmap cluster of diferential persulfidated proteins. (**A**) Heatmap of proteomic analysis from carbon-starved (S1, S3) and non-starved grown plants (S2, S4). Proteins detected in all samples were submitted to ANOVA testing (FDR 0.05) and hierarchical clustering using Perseus. Top column shows dendrogram clustering based on the biological replicates of the four sample sets and row dendrogram clustering data based on differential proteins. Values are displayed as Z-score. (**B**) Protein profiles of the proteins within each cluster. (**C**) KEGG pathway annotation from DAVID database. Graphics show the name of the pathway, dots represent the number of identified proteins (count, x axis) and the fold of enrichment (y axis), and the color code represents the –log_2_ *p* value.

Protein persulfidation profiles during carbon nutrient deprivation were analyzed in leaf tissue subjected to continuous darkness for 3 days (samples S1) and transferred for recovery for an additional 5 days (samples S3), and compared to normal growth conditions but collected in the dark (samples S2) or light (samples S4) phase of the photoperiod. Hierarchical clustering analysis correctly assigned replicates to each experimental group and showed higher correlation between control sample S2 and S4 replicas, collected after 8 h of the dark or light photoperiod, and between sample S1 and S3 replicas, which were subjected to severe or mild C starvation, respectively (Figure 3A). At least four protein clusters of persulfidated profiles were clearly distinct based on differential proteins (normalized row, Z-score). While clusters 1, 2, and 4 comprised proteins with varying persulfidation levels in the control (S2, S4) and starved plants (S1, S3), cluster 3 shows proteins whose persulfidation profiles were influenced by dark/light transition rather than by nutrient deprivation.

Cluster 1 corresponds to a group of 1405 proteins with a reduced-level profile in sample set S1; therefore, this protein group includes proteins with reduced persulfidation levels after severe carbon starvation (Figure 3B; Dataset S9). An overview of the persulfidation level of the samples, analyzed in gel using fluorescent labeling, showed that S1 samples have a more than 50% reduction in total persulfidation level compared to the rest of the samples (Figure S1). A considerable number of the proteins of this cluster, 212, representing up to 15%, are involved in protein synthesis, targeting, and degradation, with the latter being the most important group and including the autophagic protein ATG5. Pathway enrichment of the proteins within cluster 1 shows four pathways that are highlighted in association with severe nutrient and energy deprivation (Figure 3C). Three of these pathways correspond to metabolic processes that provide primary metabolites to essential energy pathways, such as the tricarboxylic acid cycle, and correspond to the “C5-branched dibasic metabolism” pathway module, the “valine, leucine and isoleucine biosynthesis” module, and the “2-oxocarboxylic acid pathway”. 

**Figure 4:**
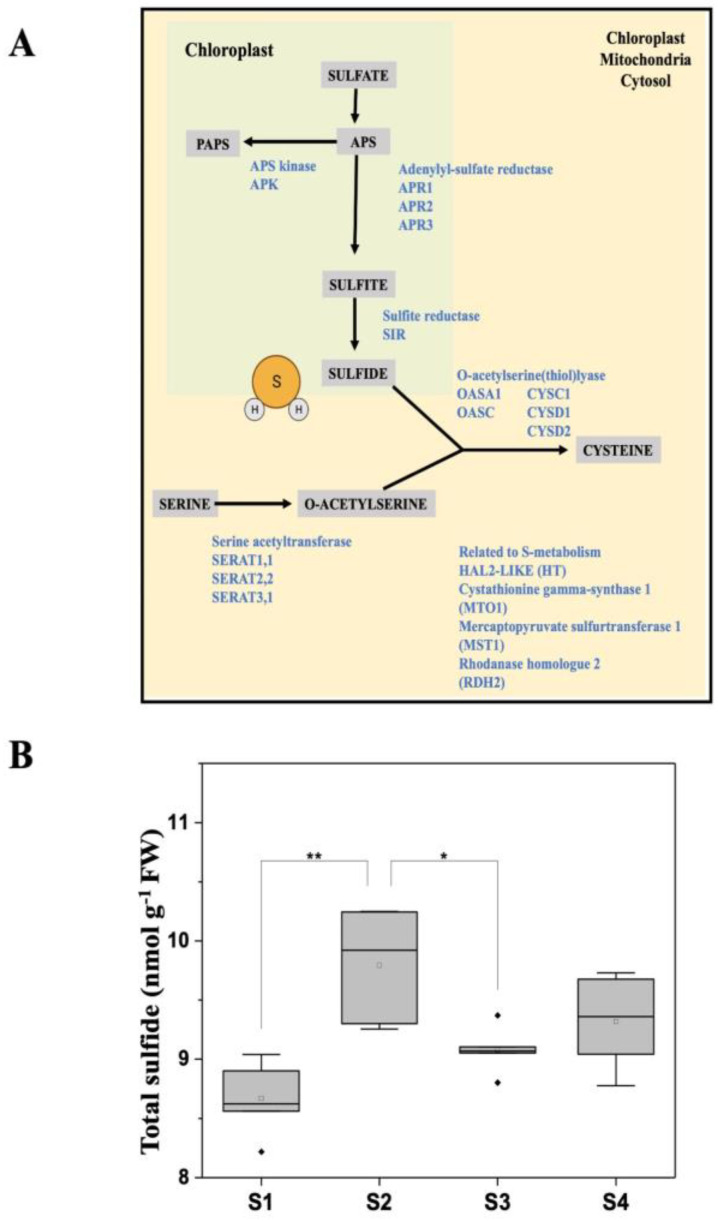
(**A**) Graphical representation of the overrepresented enzymes involved in the sulfate assimilation and cysteine biosynthesis pathways identified in cluster 1. The green panel corresponds to chloroplastic located processes and the yellow corresponds to enzymes that can be located either in the chloroplast, the mitochondria, or the cytosol compartments. APK, APS kinase; APR, APS reductase; APS, adenosine 5_-phosphosulfate; OAS/CYS, O-acetylserine(thiol)lyase; PAPS, 3_-phosphoadenosine 5_-phosphosulfate; SERAT, serine acetyltransferase; SiR, sulfite reductase. (**B**) Total sulfide content in leaf tissue. Sulfide concentration was quantified after derivation and HPLC-MS analysis in independent leaf extracts (*n* = 5). Asterisk shows significant differences, ** *p* < 0.01, * *p* < 0.05, as indicated after ANOVA and post hoc Tukey’s HSD test.

Interestingly, the fourth enriched pathway corresponded to “sulfur metabolism”, which included 19 enzymes involved in the primary sulfur assimilation and cysteine biosynthesis process that determines the synthesis of sulfide and its assimilation to cysteine (Figure 4A). Sulfide, the sulfurating species that generates persulfidation, is mainly generated in the chloroplast by ferredoxin-sulfite reductase (SIR), but sulfide incorporation into cysteine can take place in the chloroplast, mitochondria, and cytosol through the action of compartmentalized serine acetyltransferase (SERAT) and O-acetylserine(thiol)lyase (OAS/CYS) isoforms [61]. Although cluster 1 shows a significant fold enrichment in thylakoid (12,57-fold, *p*-value 7.6 × 10^−17^) and photosynthetic proteins (7.55-fold, *p*-value 1.9 × 10^−7^), the chloroplastic serine acetyltransferase (SERAT2,1) and the O-acetylserine(thiol)lyase (OASB and CS26) isoforms [21,62] do not change their level of persulfidation and, therefore, they are not represented in Figure 4A. 

To correlate the persulfidation results with the level of the sulfurating agent, we determined the total sulfide levels under the studied conditions. Sulfide levels did not vary in different phases of the growth photoperiod, although the total content of sulfide in the C-starved samples was slightly but significantly reduced by 10% (Figure 4B). However, the total average number of identified persulfidated proteins was not reduced in this sample. To verify that the changes in persulfidation levels were not due to changes in the total amount of protein that could influence the enrichment and purification process of the labeled proteins, we analyzed the protein level of one of the OASTLs identified in this subgroup using immunoblotting. Although the level of persulfidation of the cytosolic O-acetylserine(thiol)lyase A1 isoform (cytosolic OASA1) [28] is reduced under C starvation (S1), the level of this protein remains almost constant in the four sample sets as analyzed using immunoblotting (Figure S2).

**Figure 5:**
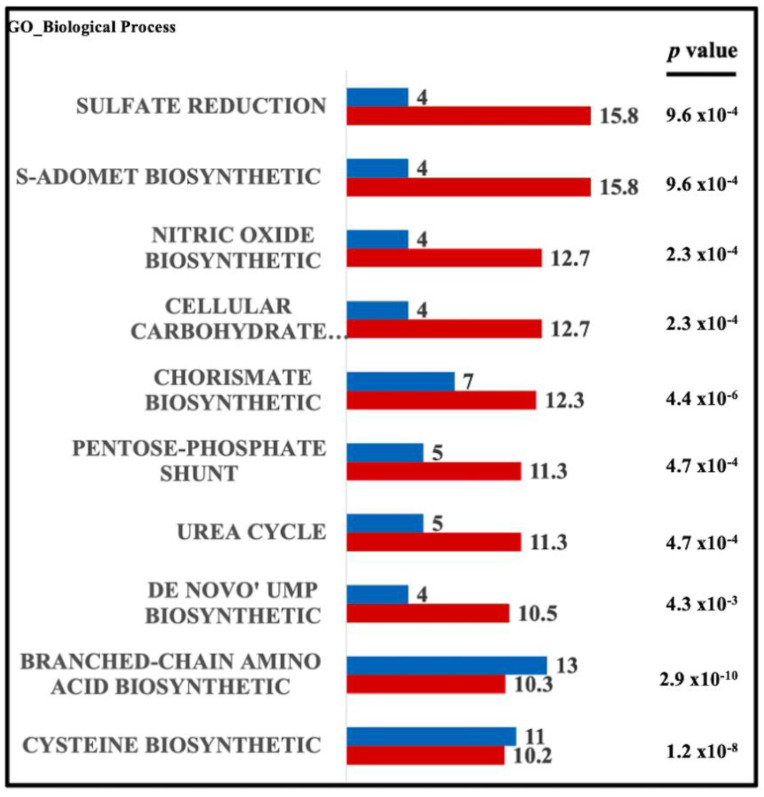
Overrepresented processes in cluster 1 based on Gene Ontology classification by biological process. Blue bars show the number of proteins identified in the process and red bars the fold of enrichment. The significance *p* value is also shown.

Further GO classification by biological process analysis of cluster 1 identified the sulfate reduction and cysteine biosynthetic pathways as significantly overrepresented, but also the nitric oxide biosynthetic route (*p*-value 0.0023), which includes the three main enzymes involved in NO production in plants, NOA1, NIA1, and NIA2, which were significantly less persulfidated under severe C starvation (S1) (Figure 5). 

When comparing the 3-day dark-adapted plants (S1) to the 5 days of recovery after the dark adaptation (S3), 2322 differentially persulfidated proteins were identified, of which 1139 were less persulfidated after recovery (Figure S3, Dataset S10). Most of the proteins of cluster 1 return to the level observed in plants growing under a normal long-day photoperiod (Figure 3B). Among these proteins with reduced levels of persulfidation, the cluster 2 subgroup is included, with 567 components (Figure 3B; Dataset S11). Enrichment analysis of this cluster showed several routes related to energy supply, such as the TCA cycle and pentose phosphate pathway (Figure 3C). After the recovery period, the plants still show a significant induction of autophagy [50]; therefore, a relevant number of the proteins of cluster 2 are related to ubiquitin-dependent protein degradation and autophagy, such as SCF coronatine insensitive 1 (COI1) ubiquitin ligase, small ubiquitin-like modifier 1 (SUMO1), and the ATG3 and ATG4b autophagic proteins, the latter of which is involved in the autophagy-dependent degradation of chloroplasts in darkened leaves [63]. 

Cluster 3 is the smallest cluster and comprises 243 proteins that showed a reduced level of persulfidation in control sample S2, collected during the night period, with GO enrichment classification within the proteasome complex, proteasomal ubiquitin protein degradation, and peptidase activity (Figure 3C). This cluster comprises most of the proteins of cluster L1 previously described (Table 2).

Finally, cluster 4 included 932 proteins with a higher persulfidation profile in the stressed sample, S1–S3, with enrichment classification classified within fatty acid beta oxidation, ribosome, peroxisome, and chloroplast envelope and stroma (Figure 3C). 

## 4. Discussion

The signaling role of H_2_S in the regulation of physio/pathological processes is already well established in mammalian systems [7,29]. In plants, several processes have already been described in which H_2_S plays an important role by regulating the function of proteins through the persulfidation of cysteine residues [1,3,64]. However, the number of processes and metabolic pathways that are regulated by sulfide need to be increased to understand the functional diversity of this gasotransmitter in plants.

The mechanism by which sulfide is capable of reacting with cysteine residues has been the subject of extensive investigation since it is not able to react directly with reduced thiols and requires the presence of an oxidant [29,65]. Therefore, H_2_S signaling and protein persulfidation need to be studied in the context of the cellular ROS levels. 

Light and the absence of light trigger different metabolic processes and affect plant growth, driving photosynthesis to obtain the energy to fix carbon and assimilate N and S nutrients [66]. Sulfur uptake is significantly higher during the day and decreases during the night period, as does S incorporation into proteins [67]; therefore, chloroplast sulfate assimilation is the major sulfide source in plant cells during the light photoperiod with a metabolic and protein synthesis fate [21,67]. However, the number of proteins susceptible to persulfidation during both the night and the light period is high, with more than four thousand identifications, suggesting that there is a high number of basally modified proteins in plant cells, although the level of persulfidation and the processes in which they are biologically involved shift substantially from one condition to the other. Previous proteomic analyses have already shown this observation that the persulfidation profile changes significantly under different physiological conditions, but the total number of modified proteins remains high [14,31,32]. This feature appears to be specific to plant cells since, because the same chemoselective labeling approach is used in animal systems, the number of identified persulfidated proteins is much lower [52,68,69]. The major difference between plant and animal systems is the source of sulfide, which depends on enzymes (CBS/CSE/3MST) that degrade cysteine-related molecules in animals, while in plants, cysteine-degrading enzymes (D/L-DES/MST) also coexist with sulfite reductase (SIR) of the sulfate assimilation pathway [20,22]. Therefore, sulfide production and protein persulfidation will depend on the level of sulfide-producing enzymes and ROS levels of the cellular compartments to allow the modification to chemically take place. 

Most of the proteins identified in the light/dark samples were common in both conditions, but 67% of the proteins did not significantly change their persulfidation levels, and only 33% were light/dark-specific or changed their modification levels depending on the photoperiod. The number of proteins that do change their persulfidation levels is twice as high in the light as in the dark and correlates with a more oxidative cellular state in the light phase. The photosynthetic electron transport chain during light reactions contributes to the production of ROS, which cause oxidative modifications of thiol groups of cysteine residues that affect enzyme activity and stability [44,70]. Therefore, under light conditions, photosynthetic-dependent ROS production and sulfide synthesis may explain the high number of proteins that increase their persulfidation levels. The GO and KEGG pathway analysis of these subsets of proteins highlights the proteasome as the one that shows the highest enrichment value, with most of the proteins corresponding to 20S proteasome subunits (Table 2). Proteasome-mediated degradation has been shown to be enhanced by cellular exposure to ROS through a switch in the predominant proteasome complex from 26S to 20S. Degradation by the 20S proteasome does not require ubiquitin tagging, and relies on the structural disorder of the protein due to oxidation, mutation, or intrinsically unfolded regions. The 20S proteasome activity has been shown to be modulated by proteasome activation through the oxidation of Cys residues into sulfenic species followed by *S*-glutathionylation. This modification changes the gating conformation to allow proteins to enter the catalytic chamber, being more effective at degrading oxidized or unfolded proteins [71,72]. Prolonged exposure to ROS also affects the proteasome itself [73]; therefore, the protective function of persulfidation against cysteine overoxidation may uncover a new physiological process regulated by H_2_S in plants. The 20S proteasome plays an important role under oxidative stress since increasing 20S proteasome activity and ubiquitin-independent degradation induce tolerance to H_2_O_2_ or methyl viologen treatments [74]. In contrast, loss mutation of the PBE1 20S beta subunit, which shows higher persulfidation levels under light conditions, or other mutations of proteasome-associated proteins subunits result in hypersensitivity to oxidative and salt stress [75,76]. 

In the absence of light and photosynthetic activity during the night, the H_2_S effects on the persulfidation profile change, with protein processing in the ER and protein folding being the more enriched GO biological processes (Table 1). The endoplasmic reticulum is the port of entry of proteins into the secretory pathway, plays a crucial role in protein synthesis, and serves as a protein-folding organelle functioning in peptide chain folding and processing [77,78]. After the entry of nascent polypeptides into the ER lumen, they promptly interact with a chaperone complex and enzymes such as protein disulfide isomerases (PDIs), cyclophilins, and UDP-glucosyltransferases to avoid premature folding and nonfunctional proteins. Many of the persulfidated proteins associated with cluster D1 correspond to enzymes of the chaperone complex and include several binding proteins (BiP) and heat shock proteins 70 (HSP70). To facilitate the formation of disulfide bonds and assist in transitioning toward a native state, this organelle provides an oxidative environment [79]. Under dark conditions, this compartment seems to be a main place of action of H_2_S since the oxidation of some Cys residues of HSPs is essential for the ATPase activity and can be modified by *S*-glutathionylation [80,81]. Recently, a role of sulfide in ER stress responses through the persulfidation of the autophagic protein ATG18a was also revealed in Arabidopsis [15]. In addition to proteins of the ER chaperone complex, other subunits of the chaperonin-containing T-complex and organellar HSP70 proteins also showed higher levels of persulfidation in the dark photoperiod, suggesting the relevant role of sulfur in the regulation of tubulin and actin folding in the cytosol and of proteins imported into the chloroplast and mitochondria [82,83,84]. 

A significant number of proteins classified within the GO biosynthesis of amino acids, mainly in valine, leucine, and isoleucine biosynthesis, also showed a higher level of persulfidation in the dark than in the light phase (Table 1). We have previously observed that the reduction in the level of persulfidation of some enzymes identified in the D2 and D3 clusters, such as CSR1, VAT1, IPMS1, or AHASS1, results in increased levels of Val, Leu, and Ile under N starvation [32]. Therefore, the reduced enzymatic capacity of persulfidated proteins may result in reduced biosynthesis of these amino acids and allow accumulation of the TCA precursors pyruvate and acetyl-CoA to maintain energy production during the night period and fatty acid biosynthesis [85,86].

H_2_S has been described to regulate physiological processes in plants during nutrient starvation, such as autophagy, through the persulfidation of the cysteine protease ATG4 [14]. Under basal growth conditions, H_2_S acts as a repressor of autophagy by repressing ATG4 activity and mutations in the enzyme DES1 that generate H_2_S within the cytosol cause constitutive autophagy induction [16,50]. Under N or C starvation, autophagy is induced to recycle cellular components; however, the exogenous addition of H_2_S represses this induction, suggesting the important role of this molecule in regulating cellular processes. In fact, recent proteomic analysis in root tissues subjected to N starvation indicates that many proteins involved in the autophagy process are susceptible to persulfidation, such as the main components of the TORC1 complex and other proteins of the autophagy core machinery, such as ATG3, ATG5, ATG7, and the already described ATG4 [32]. In this work, we provide evidence that under C starvation in leaves, there is significant reprogramming of the persulfidation profile. Therefore, under severe C starvation (S1 samples), we identified the largest cluster of proteins, cluster 1, with more than 1400 proteins, which reduced their persulfidation level compared to that under normal nutrient growth conditions (Figure 4). Within this cluster, the most enriched GO group again corresponds to BCAA biosynthesis and pathways that also include enzymes involved in their catabolism, such as branched-chain aminotransferases 3, 4, and 5. Similarly, as observed under N starvation conditions, the reduced level of persulfidation may result in the activation of biosynthesis or the degradation of these amino acids for the metabolic supply of the TCA cycle [32]. 

Interestingly, sulfur metabolism is also overrepresented in cluster 1, with almost all the enzymes of the sulfate assimilation and cysteine biosynthesis pathways (Figure 5). Sulfide, the sulfurating species that generates persulfidation, is mainly generated in the chloroplast by ferredoxin-sulfite reductase (SIR), but sulfide incorporation into cysteine takes place mainly in the cytosol [21,61]. Although cluster 1 shows a significant fold enrichment of chloroplastic proteins, the chloroplastic serine acetyltransferase (SERAT2,1) and the *O*-acetylserine(thiol)lyase (OASB and CS26) isoforms [21,62] do not change their level of persulfidation. The total level of the O-acetylserine(thiol)lyase A1 isoform (cytosolic OASA1) [28], the main enzyme that incorporates sulfide into cysteine, remained almost constant in the four sample sets; therefore, the reduced level of persulfidation of the cytosolic (SERAT1,1, SERAT3,1, OASA1, CYSD1, CYSD2) and mitochondrial (OASC, CYSC1) isoforms seems to be unrelated to a reduction in chloroplast sulfide biosynthesis under dark conditions. Alternatively, the change in protein persulfidation levels could affect the enzymatic activity levels of these proteins and, therefore, alter chloroplast sulfide incorporation and the total sulfide content.

Although autophagy plays a significant role during the nutrient stress period, we only detected a change in the persulfidation level of the ATG5 protein in cluster 1, not in other ATG proteins, as observed under N starvation in root tissues [32].

C starvation after recovery again shifted the persulfide proteome to highlight a cluster of 567 proteins with reduced levels of persulfidation (cluster 2, Figure 4). The most enriched and abundant protein groups corresponded to energy-related pathways such as the TCA cycle and the pentose phosphate pathway (PPP). The glucose 6-phosphate (G6P) molecule is the first step of glycolysis, and can be further broken down into the process of glycolysis to provide energy (ATP) or shunted into the pentose phosphate pathway (PPP) to provide reducing equivalents (NADPH) and pentose sugars as precursors of nucleotide and amino acid biosynthesis. The fate of glucose in glycolysis or the PPP is regulated by nutrient concentration or the environmental conditions, largely determined by the availability of oxygen and the energy needs of the cell [87]. The reduced level of persulfidation of glucose-6-phosphate dehydrogenase, which catalyzes the first step in the oxidative PPP, suggests a new functional role of H_2_S in the regulation of metabolic processes under nutrient deprivation or oxidative stress to direct the production of NADPH. Cysteine persulfidation has a broad effect on protein functionality both by activating [12,34,35] and inhibiting their function [14,15]. A detailed study of the effect of persulfidation on the revealed target proteins will be necessary in the future to deepen understanding of the signaling role of H_2_S in the identified physiological processes. 

## 5. Conclusions

The analysis of the persulfidation profiles carried out in this work highlights new metabolic and biological processes regulated by H_2_S in the context of two essential physiological situations in plants, the photoperiod phase of growth and carbon nutrition/starvation, which are closely linked to light conditions. The quantitative analysis of the protein persulfidation level highlights new processes that have not previously been identified in which H_2_S molecules may play a signaling or protective role, such as protein processing and folding in the ER during the dark phase of the day, proteasomal protein catabolism, pentose-phosphate shunt in the light phase, and sulfur assimilation during carbon deprivation, among others. Further individual studies of these processes and the identified protein targets will be necessary to specify the effect of persulfidation on the activation or inhibition of the processes, since both alternatives have been previously described. 

## Figures and Tables

**Table 1:** Functional Annotation Clustering by GO_Biological Function and KEGG pathway of the proteins only present or more persulfidated in dark collected sample (S2).

**Annotation Cluster D1**				
**Enrichment Score: 6.6**	**Term**	**Count**	***p* Value**	**Benjamini**
GOTERM_BP	Protein folding	34	1.70 × 10^−13^	6.10 × 10^−11^
GOTERM_BP	Response to heat	16	9.50 × 10^−6^	1.30 × 10^−3^
KEGG_PATHWAY	Protein processing in ER	19	9.70 × 10^−3^	1.30 × 10^−1^
**Annotation Cluster D2**				
**Enrichment Score: 2.13**	**Term**	**Count**	***p* Value**	**Benjamini**
KEGG_PATHWAY	Biosynthesis of amino acids	23	2.30 × 10^−3^	6.40 × 10^−2^
KEGG_PATHWAY	Biosynthesis secondary metabolites	64	3.50 × 10^−3^	6.40 × 10^−2^
KEGG_PATHWAY	Metabolic pathways	91	1.70 × 10^−1^	7.10 × 10^−1^
**Annotation Cluster D3**				
**Enrichment Score: 2.05**	**Term**	**Count**	***p* Value**	**Benjamini**
GOTERM_BP	Branched-chain AA biosynthetic process	6	9.90 × 10^−5^	7.80 × 10^−3^
KEGG_PATHWAY	Valine, leucine, isoleucine biosynthesis	6	3.10 × 10^−3^	6.40 × 10^−2^
KEGG_PATHWAY	2-Oxocarboxylic acid metabolism	9	1.80 × 10^−2^	1.60 × 10^−1^

**Table 2:** Functional Annotation Clustering by GO_Biological Function and KEGG pathway of the proteins only present or more persulfidated in light collected sample (S4).

**Annotation Cluster L1**				
**Enrichment Score: 4.68**	**Term**	**Count**	***p* Value**	**Benjamini**
GOTERM_BP	Proteasomal Ubiq-independent protein catabolic process	18	9.50 × 10^−19^	4.70 × 10^−16^
GOTERM_BP	Proteasomal protein catabolic process	15	6.10 × 10^−14^	2.00 × 10^−11^
KEGG_PATHWAY	Proteasome	20	1.30 × 10^−7^	1.20 × 10^−5^
GOTERM_BP	Ubiq-dependent protein catabolic process	23	2.10 × 10^−2^	3.40 × 10^−1^
**Annotation Cluster L2**				
**Enrichment Score: 3.02**	**Term**	**Count**	***p* Value**	**Benjamini**
KEGG_PATHWAY	Glutathione metabolism	34	1.10 × 10^−12^	2.90 × 10^−10^
GOTERM_BP	Glutathione metabolic process	13	9.40 × 10^−6^	7.90 × 10^−4^
GOTERM_BP	Toxin catabolic process	10	6.70 × 10^−5^	3.70 × 10^−3^
**Annotation Cluster L3**				
**Enrichment Score: 2.21**	**Term**	**Count**	***p* Value**	**Benjamini**
GOTERM_BP	Nucleosome assembly	10	2.10 × 10^−5^	1.50 × 10^−3^
GOTERM_BP	Somatic cell DNA recombination	5	2.60 × 10^−4^	1.20 × 10^−2^
GOTERM_BP	Double-strand break repair via homologous recombination	5	2.70 × 10^−1^	1.00
**Annotation Cluster L4**				
**Enrichment Score: 1.96**	**Term**	**Count**	***p* Value**	**Benjamin**
GOTERM_BP	Glycolytic process	10	1.00 × 10^−3^	4.00 × 10^−2^
KEGG_PATHWAY	Glycolysis/Gluconeogenesis	20	2.60 × 10^−3^	3.70 × 10^−2^
GOTERM_BP	Pentose-phosphate shunt	6	2.60 × 10^−3^	9.10 × 10^−2^
GOTERM_BP	Gluconeogenesis	5	1.60 × 10^−2^	2.90 × 10^−1^

## Data Availability

The mass spectrometry proteomics data have been deposited in the ProteomeXchange Consortium via the PRIDE partner repository with the dataset identifier PXD024061.

## Supplementary Materials

The following supporting information can be downloaded at: https://www.mdpi.com/article/10.3390/antiox12040789/s1, Dataset S1; Dataset S2; Dataset S3; Dataset S4; Dataset S5; Dataset S6; Dataset S7; Dataset S8; Dataset S9; Dataset S10; Dataset S11; Figure S1: Protein persulfidation profile; Figure S2: Protein immunoblotting detection of the cytosolic OASA1 isoform; Figure S3: Hierarchical clustering of persulfidated proteins in severe C-starved samples and C-starved samples after recovery; Table S1: Identified proteins in cluster D1 related to the GO_BF term protein folding; Table S2: Identified proteins in cluster D2 related to carbon metabolism; Table S3: Identified proteins in light clusters according to GO_BF and KEGG classifications.

## References

[1] Aroca A., Zhang J., Xie Y., Romero L.C., Gotor C. (2021). Hydrogen sulfide signaling in plant adaptations to adverse conditions: Molecular mechanisms. J. Exp. Bot..

[2] Aroca A., Gotor C., Bassham D.C., Romero L.C. (2020). Hydrogen Sulfide: From a Toxic Molecule to a Key Molecule of Cell Life. Antioxidants.

[3] Zhou M., Zhou H., Shen J., Zhang Z., Gotor C., Romero L.C., Yuan X., Xie Y. (2021). H2S action in plant life cycle. Plant Growth Regul..

[4] Walsh B.J.C., Giedroc D.P. (2020). H(2)S and reactive sulfur signaling at the host-bacterial pathogen interface. J. Biol. Chem..

[5] Toliver-Kinsky T., Cui W., Toro G., Lee S.J., Shatalin K., Nudler E., Szabo C. (2019). H2S, a Bacterial Defense Mechanism against the Host Immune Response. Infect. Immun..

[6] Petrovic D., Kouroussis E., Vignane T., Filipovic M.R. (2021). The Role of Protein Persulfidation in Brain Aging and Neurodegeneration. Front. Aging Neurosci..

[7] Yadav P.K., Martinov M., Vitvitsky V., Seravalli J., Wedmann R., Filipovic M.R., Banerjee R. (2016). Biosynthesis and Reactivity of Cysteine Persulfides in Signaling. J. Am. Chem. Soc..

[8] Xiao Q., Ying J., Xiang L., Zhang C. (2018). The biologic effect of hydrogen sulfide and its function in various diseases. Medicine.

[9] Arif M.S., Yasmeen T., Abbas Z., Ali S., Rizwan M., Aljarba N.H., Alkahtani S., Abdel-Daim M.M. (2021). Role of Exogenous and Endogenous Hydrogen Sulfide (H2S) on Functional Traits of Plants Under Heavy Metal Stresses: A Recent Perspective. Front. Plant Sci..

[10] Zhou H., Zhou Y., Zhang F., Guan W., Su Y., Yuan X., Xie Y. (2021). Persulfidation of Nitrate Reductase 2 Is Involved in l-Cysteine Desulfhydrase-Regulated Rice Drought Tolerance. Int. J. Mol. Sci..

[11] Scuffi D., Álvarez C., Laspina N., Gotor C., Lamattina L., García-Mata C. (2014). Hydrogen Sulfide Generated by l-Cysteine Desulfhydrase Acts Upstream of Nitric Oxide to Modulate Abscisic Acid-Dependent Stomatal Closure. Plant Physiol..

[12] Shen J., Zhang J., Zhou M., Zhou H., Cui B., Gotor C., Romero L.C., Fu L., Yang J., Foyer C.H. (2020). Persulfidation-based Modification of Cysteine Desulfhydrase and the NADPH Oxidase RBOHD Controls Guard Cell Abscisic Acid Signaling. Plant Cell.

[13] Zhou H., Chen Y., Zhai F., Zhang J., Zhang F., Yuan X., Xie Y. (2020). Hydrogen sulfide promotes rice drought tolerance via reestablishing redox homeostasis and activation of ABA biosynthesis and signaling. Plant Physiol. Biochem..

[14] Laureano-Marin A.M., Aroca A., Perez-Perez M.E., Yruela I., Jurado-Flores A., Moreno I., Crespo J.L., Romero L.C., Gotor C. (2020). Abscisic Acid-Triggered Persulfidation of the Cys Protease ATG4 Mediates Regulation of Autophagy by Sulfide. Plant Cell.

[15] Aroca A., Yruela I., Gotor C., Bassham D.C. (2021). Persulfidation of ATG18a regulates autophagy under ER stress in Arabidopsis. Proc. Natl. Acad. Sci. USA.

[16] Laureano-Marin A.M., Moreno I., Romero L.C., Gotor C. (2016). Negative Regulation of Autophagy by Sulfide Is Independent of Reactive Oxygen Species. Plant Physiol..

[17] Zhou M., Zhang J., Zhou H., Zhao D., Duan T., Wang S., Yuan X., Xie Y. (2022). Hydrogen Sulfide-Linked Persulfidation Maintains Protein Stability of ABSCISIC ACID-INSENSITIVE 4 and Delays Seed Germination. Int. J. Mol. Sci..

[18] Zhou Z.H., Wang Y., Ye X.Y., Li Z.G. (2018). Signaling Molecule Hydrogen Sulfide Improves Seed Germination and Seedling Growth of Maize (*Zea mays* L.) Under High Temperature by Inducing Antioxidant System and Osmolyte Biosynthesis. Front. Plant Sci..

[19] Kou N., Xiang Z., Cui W., Li L., Shen W. (2018). Hydrogen sulfide acts downstream of methane to induce cucumber adventitious root development. J. Plant Physiol..

[20] Cao X., Ding L., Xie Z.Z., Yang Y., Whiteman M., Moore P.K., Bian J.S. (2019). A Review of Hydrogen Sulfide Synthesis, Metabolism, and Measurement: Is Modulation of Hydrogen Sulfide a Novel Therapeutic for Cancer?. Antioxid. Redox Signal..

[21] Takahashi H., Kopriva S., Giordano M., Saito K., Hell R. (2011). Sulfur assimilation in photosynthetic organisms: Molecular functions and regulations of transporters and assimilatory enzymes. Annu. Rev. Plant Biol..

[22] Gotor C., Garcia I., Aroca A., Laureano-Marin A.M., Arenas-Alfonseca L., Jurado-Flores A., Moreno I., Romero L.C. (2019). Signaling by hydrogen sulfide and cyanide through post-translational modification. J. Exp. Bot..

[23] Alvarez C., Calo L., Romero L.C., Garcia I., Gotor C. (2010). An O-acetylserine(thiol)lyase homolog with L-cysteine desulfhydrase activity regulates cysteine homeostasis in Arabidopsis. Plant Physiol..

[24] Jin Z., Shen J., Qiao Z., Yang G., Wang R., Pei Y. (2011). Hydrogen sulfide improves drought resistance in Arabidopsis thaliana. Biochem. Biophys. Res. Commun..

[25] Alvarez C., García I., Romero L.C., Gotor C. (2012). Mitochondrial Sulfide Detoxification Requires a Functional Isoform O-Acetylserine(thiol)lyase C in Arabidopsis thaliana. Mol. Plant.

[26] Garcia I., Castellano J.M., Vioque B., Solano R., Gotor C., Romero L.C. (2010). Mitochondrial beta-cyanoalanine synthase is essential for root hair formation in Arabidopsis thaliana. Plant Cell.

[27] Wang Z., He F., Mu Y., Zhang L., Liu Z., Liu D., Yang J., Jin Z., Pei Y. (2022). Identification and functional characterization of a cystathionine β-lyase (CBL) enzyme for H(2)S production in Arabidopsis thaliana. Plant Physiol. Biochem..

[28] Dominguez-Solis J.R., Gutierrez-Alcala G., Vega J.M., Romero L.C., Gotor C. (2001). The cytosolic O-acetylserine(thiol)lyase gene is regulated by heavy metals and can function in cadmium tolerance. J. Biol. Chem..

[29] Filipovic M.R., Zivanovic J., Alvarez B., Banerjee R. (2018). Chemical Biology of H2S Signaling through Persulfidation. Chem. Rev..

[30] Aroca Á., Serna A., Gotor C., Romero L.C. (2015). S-Sulfhydration: A Cysteine Posttranslational Modification in Plant Systems. Plant Physiol..

[31] Aroca A., Benito J.M., Gotor C., Romero L.C. (2017). Persulfidation proteome reveals the regulation of protein function by hydrogen sulfide in diverse biological processes in Arabidopsis. J. Exp. Bot..

[32] Jurado-Flores A., Romero L.C., Gotor C. (2021). Label-Free Quantitative Proteomic Analysis of Nitrogen Starvation in Arabidopsis Root Reveals New Aspects of H2S Signaling by Protein Persulfidation. Antioxidants.

[33] Aroca A., Schneider M., Scheibe R., Gotor C., Romero L.C. (2017). Hydrogen Sulfide Regulates the Cytosolic/Nuclear Partitioning of Glyceraldehyde-3-Phosphate Dehydrogenase by Enhancing its Nuclear Localization. Plant Cell Physiol..

[34] Chen S., Jia H., Wang X., Shi C., Wang X., Ma P., Wang J., Ren M., Li J. (2020). Hydrogen Sulfide Positively Regulates Abscisic Acid Signaling through Persulfidation of SnRK2.6 in Guard Cells. Mol. Plant.

[35] Zhou M., Zhang J., Shen J., Zhou H., Zhao D., Gotor C., Romero L.C., Fu L., Li Z., Yang J. (2021). Hydrogen sulfide-linked persulfidation of ABI4 controls ABA responses through the transactivation of MAPKKK18 in Arabidopsis. Mol. Plant.

[36] Filipovic M.R., Moore P.K., Whiteman M. (2015). Persulfidation (S-sulfhydration) and H2S. Chemistry, Biochemistry and Pharmacology of Hydrogen Sulfide.

[37] Vasas A., Doka E., Fabian I., Nagy P. (2015). Kinetic and thermodynamic studies on the disulfide-bond reducing potential of hydrogen sulfide. Nitric Oxide.

[38] Cuevasanta E., Lange M., Bonanata J., Coitiño E.L., Ferrer-Sueta G., Filipovic M.R., Alvarez B. (2015). Reaction of Hydrogen Sulfide with Disulfide and Sulfenic Acid to Form the Strongly Nucleophilic Persulfide. J. Biol. Chem..

[39] Zivanovic J., Kouroussis E., Kohl J.B., Adhikari B., Bursac B., Schott-Roux S., Petrovic D., Miljkovic J.L., Thomas-Lopez D., Jung Y. (2019). Selective Persulfide Detection Reveals Evolutionarily Conserved Antiaging Effects of S-Sulfhydration. Cell Metab..

[40] Doka E., Ida T., Dagnell M., Abiko Y., Luong N.C., Balog N., Takata T., Espinosa B., Nishimura A., Cheng Q. (2020). Control of protein function through oxidation and reduction of persulfidated states. Sci. Adv..

[41] Foyer C.H., Noctor G., Buchanan B., Dietz K.J., Pfannschmidt T. (2009). Redox Regulation in Photosynthetic Organisms: Signaling, Acclimation, and Practical Implications. Antioxid. Redox Signal..

[42] Suzuki N., Koussevitzky S., Mittler R., Miller G. (2012). ROS and redox signalling in the response of plants to abiotic stress. Plant Cell Environ..

[43] Noctor G., Reichheld J.-P., Foyer C.H. (2018). ROS-related redox regulation and signaling in plants. Semin. Cell Dev. Biol..

[44] Buchanan B.B., Balmer Y. (2005). Redox regulation: A broadening horizon. Annu. Rev. Plant Biol..

[45] Zimmer D., Swart C., Graf A., Arrivault S., Tillich M., Proost S., Nikoloski Z., Stitt M., Bock R., Mühlhaus T. (2021). Topology of the redox network during induction of photosynthesis as revealed by time-resolved proteomics in tobacco. Sci. Adv..

[46] Yu J., Li Y., Qin Z., Guo S., Li Y., Miao Y., Song C., Chen S., Dai S. (2020). Plant Chloroplast Stress Response: Insights from Thiol Redox Proteomics. Antioxid. Redox Signal..

[47] Yang J., Carroll K.S., Liebler D.C. (2016). The Expanding Landscape of the Thiol Redox Proteome. Mol. Cell. Proteom..

[48] Zaffagnini M., Fermani S., Marchand C.H., Costa A., Sparla F., Rouhier N., Geigenberger P., Lemaire S.D., Trost P. (2019). Redox Homeostasis in Photosynthetic Organisms: Novel and Established Thiol-Based Molecular Mechanisms. Antioxid. Redox Signal..

[49] Bermudez M.A., Galmes J., Moreno I., Mullineaux P.M., Gotor C., Romero L.C. (2012). Photosynthetic adaptation to length of day is dependent on S-sulfocysteine synthase activity in the thylakoid lumen. Plant Physiol..

[50] Alvarez C., Garcia I., Moreno I., Perez-Perez M.E., Crespo J.L., Romero L.C., Gotor C. (2012). Cysteine-generated sulfide in the cytosol negatively regulates autophagy and modulates the transcriptional profile in Arabidopsis. Plant Cell.

[51] Tan B., Jin S., Sun J., Gu Z., Sun X., Zhu Y., Huo K., Cao Z., Yang P., Xin X. (2017). New method for quantification of gasotransmitter hydrogen sulfide in biological matrices by LC-MS/MS. Sci. Rep..

[52] Latorre J., Aroca A., Fernandez-Real J.M., Romero L.C., Moreno-Navarrete J.M. (2022). The Combined Partial Knockdown of CBS and MPST Genes Induces Inflammation, Impairs Adipocyte Function-Related Gene Expression and Disrupts Protein Persulfidation in Human Adipocytes. Antioxidants.

[53] Bradford M.M. (1976). A rapid and sensitive method for the quantitation of microgram quantities of protein utilizing the principle of protein-dye binding. Anal. Biochem..

[54] Cox J., Mann M. (2008). MaxQuant enables high peptide identification rates, individualized p.p.b.-range mass accuracies and proteome-wide protein quantification. Nat. Biotechnol..

[55] Tyanova S., Temu T., Sinitcyn P., Carlson A., Hein M.Y., Geiger T., Mann M., Cox J. (2016). The Perseus computational platform for comprehensive analysis of (prote)omics data. Nat. Methods.

[56] Krstic J., Reinisch I., Schindlmaier K., Galhuber M., Berger N., Kupper N., Moyschewitz E., Auer M., Michenthaler H., Nössing C. (2021). Fasting reverses drug-resistance in hepatocellular carcinoma through p53-dependent metabolic synergism. bioRxiv.

[57] Vizcaino J.A., Csordas A., del-Toro N., Dianes J.A., Griss J., Lavidas I., Mayer G., Perez-Riverol Y., Reisinger F., Ternent T. (2016). 2016 update of the PRIDE database and its related tools. Nucleic Acids Res..

[58] Klie S., Nikoloski Z. (2012). The Choice between MapMan and Gene Ontology for Automated Gene Function Prediction in Plant Science. Front. Genet..

[59] Jiao X., Sherman B.T., Huang D.W., Stephens R., Baseler M.W., Lane H.C., Lempicki R.A. (2012). DAVID-WS: A stateful web service to facilitate gene/protein list analysis. Bioinformatics.

[60] Aroca A., Jurado-Flores A., Filipovic M.R., Gotor C., Romero L.C. (2022). Detection of protein persulfidation in plants by the dimedone switch method. Methods Enzymol..

[61] Garcia I., Gotor C., Romero L.C., D’Mello J.P.F. (2015). Cysteine homeostasis. Amino Acids in Higher Plants.

[62] Bermudez M.A., Paez-Ochoa M.A., Gotor C., Romero L.C. (2010). Arabidopsis S-sulfocysteine synthase activity is essential for chloroplast function and long-day light-dependent redox control. Plant Cell.

[63] Wada S., Ishida H., Izumi M., Yoshimoto K., Ohsumi Y., Mae T., Makino A. (2009). Autophagy plays a role in chloroplast degradation during senescence in individually darkened leaves. Plant Physiol..

[64] Gotor C., Aroca A., Romero L.C. (2022). Persulfidation is the mechanism underlying sulfide-signaling of autophagy. Autophagy.

[65] Cuevasanta E., Moller M.N., Alvarez B. (2017). Biological chemistry of hydrogen sulfide and persulfides. Arch. Biochem. Biophys..

[66] Eberhard S., Finazzi G., Wollman F.A. (2008). The dynamics of photosynthesis. Annu. Rev. Genet..

[67] Samuilov S., Brilhaus D., Rademacher N., Flachbart S., Arab L., Alfarraj S., Kuhnert F., Kopriva S., Weber A.P.M., Mettler-Altmann T. (2018). The Photorespiratory BOU Gene Mutation Alters Sulfur Assimilation and Its Crosstalk With Carbon and Nitrogen Metabolism in Arabidopsis thaliana. Front. Plant Sci..

[68] Comas F., Latorre J., Ortega F., Arnoriaga Rodriguez M., Kern M., Lluch A., Ricart W., Bluher M., Gotor C., Romero L.C. (2021). Activation of Endogenous H2S Biosynthesis or Supplementation with Exogenous H2S Enhances Adipose Tissue Adipogenesis and Preserves Adipocyte Physiology in Humans. Antioxid. Redox Signal..

[69] Fu L., Liu K., He J., Tian C., Yu X., Yang J. (2020). Direct Proteomic Mapping of Cysteine Persulfidation. Antioxid. Redox Signal..

[70] Hajiboland R., Ahmad P. (2014). Chapter 1—Reactive Oxygen Species and Photosynthesis. Oxidative Damage to Plants.

[71] Silva G.M., Netto L.E., Simões V., Santos L.F., Gozzo F.C., Demasi M.A., Oliveira C.L., Bicev R.N., Klitzke C.F., Sogayar M.C. (2012). Redox control of 20S proteasome gating. Antioxid. Redox Signal..

[72] Lefaki M., Papaevgeniou N., Chondrogianni N. (2017). Redox regulation of proteasome function. Redox Biol..

[73] Reinheckel T., Ullrich O., Sitte N., Grune T. (2000). Differential Impairment of 20S and 26S Proteasome Activities in Human Hematopoietic K562 Cells during Oxidative Stress. Arch. Biochem. Biophys..

[74] Kurepa J., Toh E.A., Smalle J.A. (2008). 26S proteasome regulatory particle mutants have increased oxidative stress tolerance. Plant J..

[75] Han J.-J., Yang X., Wang Q., Tang L., Yu F., Huang X., Wang Y., Liu J.-X., Xie Q. (2019). The β5 subunit is essential for intact 26S proteasome assembly to specifically promote plant autotrophic growth under salt stress. New Phytol..

[76] Bonea D., Noureddine J., Gazzarrini S., Zhao R. (2021). Oxidative and salt stresses alter the 26S proteasome holoenzyme and associated protein profiles in Arabidopsis thaliana. BMC Plant Biol..

[77] Strasser R. (2018). Protein Quality Control in the Endoplasmic Reticulum of Plants. Annu. Rev. Plant Biol..

[78] Galili G., Sengupta-Gopalan C., Ceriotti A. (1998). The endoplasmic reticulum of plant cells and its role in protein maturation and biogenesis of oil bodies. Plant Mol. Biol..

[79] Margittai É., Enyedi B., Csala M., Geiszt M., Bánhegyi G. (2015). Composition of the redox environment of the endoplasmic reticulum and sources of hydrogen peroxide. Free Radic. Biol. Med..

[80] Miyata Y., Rauch J.N., Jinwal U.K., Thompson A.D., Srinivasan S., Dickey C.A., Gestwicki J.E. (2012). Cysteine reactivity distinguishes redox sensing by the heat-inducible and constitutive forms of heat shock protein 70. Chem. Biol..

[81] Yang J., Zhang H., Gong W., Liu Z., Wu H., Hu W., Chen X., Wang L., Wu S., Chen C. (2020). S-Glutathionylation of human inducible Hsp70 reveals a regulatory mechanism involving the C-terminal α-helical lid. J. Biol. Chem..

[82] Ahn H.K., Yoon J.T., Choi I., Kim S., Lee H.S., Pai H.S. (2019). Functional characterization of chaperonin containing T-complex polypeptide-1 and its conserved and novel substrates in Arabidopsis. J. Exp. Bot..

[83] Trösch R., Mühlhaus T., Schroda M., Willmund F. (2015). ATP-dependent molecular chaperones in plastids—More complex than expected. Biochim. Biophys. Acta.

[84] Padidam M., Reddy V.S., Beachy R.N., Fauquet C.M. (1999). Molecular characterization of a plant mitochondrial chaperone GrpE. Plant Mol. Biol..

[85] Xing A., Last R.L. (2017). A Regulatory Hierarchy of the Arabidopsis Branched-Chain Amino Acid Metabolic Network. Plant Cell.

[86] Binder S. (2010). Branched-Chain Amino Acid Metabolism in Arabidopsis thaliana. Arab. Book/Am. Soc. Plant Biol..

[87] Stincone A., Prigione A., Cramer T., Wamelink M.M., Campbell K., Cheung E., Olin-Sandoval V., Grüning N.M., Krüger A., Tauqeer Alam M. (2015). The return of metabolism: Biochemistry and physiology of the pentose phosphate pathway. Biol. Rev. Camb. Philos. Soc..

