# Supplementary material for: Proteome Dynamics of Persulfidation in Leaf Tissue under Light/Dark Conditions and Carbon Deprivation
Source: Antioxidants (Basel). 2023 Mar 23;12(4):789. doi: 10.3390/antiox12040789 (PMC10135009; doi:10.3390/antiox12040789)
Supplement: Supplementary file 1 [file antioxidants-12-00789-s001.zip › Table S1.pdf]

Table S1. Identified proteins in Cluster D1 related with the GO_BF term, protein folding.

AGI_ID	Gene name
AT3G60740	ARM repeat superfamily protein (TTN1)
AT2G04030	Chaperone protein htpG family protein (CR88)
AT2G22360	DNAJ heat shock family protein
AT3G62600	DNAJ heat shock family protein (ATERDJ3B)
AT3G09440	Heat shock protein 70 (Hsp 70) family protein
AT5G42020	Heat shock protein 70 (Hsp 70) family protein (BIP2)
AT1G09080	Heat shock protein 70 (Hsp 70) family protein (BIP3)
AT5G02490	Heat shock protein 70 (Hsp 70) family protein (Hsp70-2)
AT3G20050	T-complex protein 1 alpha subunit (TCP-1)
AT1G24510	TCP-1/cpn60 chaperonin family protein
AT3G02530	TCP-1/cpn60 chaperonin family protein
AT3G03960	TCP-1/cpn60 chaperonin family protein
AT3G11830	TCP-1/cpn60 chaperonin family protein
AT3G18190	TCP-1/cpn60 chaperonin family protein
AT5G20890	TCP-1/cpn60 chaperonin family protein
AT5G26360	TCP-1/cpn60 chaperonin family protein
AT1G56340	Calreticulin 1a (CRT1a)
AT1G09210	Calreticulin 1b (CRT1b)
AT1G08450	Calreticulin 3 (CRT3)
AT1G55490	Chaperonin 60 beta CPN60B)
AT2G44650	Chloroplast chaperonin 10(CHL-CPN10)
AT4G24280	Chloroplast heat shock protein 70-1(cpHsc70-1)
AT5G49910	Chloroplast heat shock protein 70-2(cpHsc70-2)
AT5G48030	gametophytic factor 2 (GFA2)
AT5G02500	Heat shock cognate protein 70-1(HSC70-1)
AT5G28540	Heat shock protein 70 (Hsp 70) family protein (BIP1)
AT1G79930	Heat shock protein 91 (HSP91)
AT4G37910	mitochondrial heat shock protein 70-1 (mtHsc70-1)
AT5G58710	rotamase CYP 7 (ROC7)
AT5G39950	Thioredoxin 2 (TRX2)
AT5G16400	Thioredoxin F2 TRXF2)
AT1G45145	Thioredoxin H-type 5 (TRX5)
AT2G47470	Thioredoxin family protein (UNE5)
AT4G03520	Thioredoxin superfamily protein (ATHM2)
AT1G77510	PDI-like 1-2 (PDIL1-2)
