# Supplementary material for: Proteome Dynamics of Persulfidation in Leaf Tissue under Light/Dark Conditions and Carbon Deprivation
Source: Antioxidants (Basel). 2023 Mar 23;12(4):789. doi: 10.3390/antiox12040789 (PMC10135009; doi:10.3390/antiox12040789)
Supplement: Supplementary file 1 [file antioxidants-12-00789-s001.zip › Table S2.pdf]

Table S2. Identified proteins in cluster D2 related with Carbon Metabolism.

AGI_ID	Gene name
Carbon metabolism	
AT3G55410	2-oxoglutarate dehydrogenase, E1 component
AT1G12230	Aldolase superfamily protein
AT1G65930	cICDH; cytosolic NADP+-dependent isocitrate dehydrogenase
AT1G34430	EMB3003; 2-oxoacid dehydrogenases acyltransferase family protein
AT1G43670	FBP; Inositol monophosphatase family protein
AT1G23310	GGT1; glutamate:glyoxylate aminotransferase
AT1G09780	iPGAM1; Phosphoglycerate mutase, 2,3-bisphosphoglycerate-independent
AT3G25860	LTA2; 2-oxoacid dehydrogenases acyltransferase family protein
AT1G48030	mtLPD1; mitochondrial lipoamide dehydrogenase 1
AT1G01090	PDH-E1_ALPHA; pyruvate dehydrogenase E1 alpha
ATCG00490	RBCL; ribulose bisphosphate carboxylase larga chain
AT1G67090	RBCS1A; ribulose bisphosphate carboxylase small chain 1A
AT5G38430	RBCS1B; Ribulose bisphosphate carboxylase (small chain) family protein
AT5G38410	RBCS3B; Ribulose bisphosphate carboxylase (small chain) family protein
AT5G66760	SDH1-1; succinate dehydrogenase 1-1
AT3G27380	SDH2-1; succinate dehydrogenase 2-1
AT4G37930	SHM1; serine transhydroxymethyltransferase 1
AT4G13930	SHM4; serine hydroxymethyltransferase 4
AT3G60750	Transketolase
TCA cycle	
AT3G55410	2-oxoglutarate dehydrogenase, E1 component
AT1G65930	cICDH; cytosolic NADP+-dependent isocitrate dehydrogenase
AT1G34430	EMB3003; 2-oxoacid dehydrogenases acyltransferase family protein
AT3G25860	LTA2; 2-oxoacid dehydrogenases acyltransferase family protein
AT1G48030	mtLPD1; mitochondrial lipoamide dehydrogenase 1
AT1G01090	PDH-E1_ALPHA; pyruvate dehydrogenase E1 alpha
AT5G66760	SDH1-1; succinate dehydrogenase 1-1
AT3G27380	SDH2-1; succinate dehydrogenase 2-1
Glycolysis	
AT1G34430	2-oxoacid dehydrogenases acyltransferase family protein
AT1G43670	FBP; Inositol monophosphatase family protein
AT3G47800	Galactose mutarotase-like superfamily protein
AT1G09780	iPGAM1; Phosphoglycerate mutase, 2,3-bisphosphoglycerate-independent
AT3G25860	LTA2; 2-oxoacid dehydrogenases acyltransferase family protein
AT1G48030	mtLPD1; mitochondrial lipoamide dehydrogenase 1
AT5G54960	PDC2; pyruvate decarboxylase-2
AT1G01090	PDH-E1_ALPHA; pyruvate dehydrogenase E1 alpha
Oxidative phosphorylation	
AT5G08680	ATP synthase alpha/beta family protein nit (DET3)
AT1G51650	ATP synthase epsilon chain
AT2G33040	ATP3; gamma subunit of Mt ATP synthase
AT5G47030	ATPase, F1 complex, delta/epsilon subunit
AT4G09650	ATPD; F-type H+-transporting ATPase subunit delta
AT1G12840	DET3; vacuolar ATP synthase subunit C (VATC)
AT5G66760	SDH1-1; succinate dehydrogenase 1-1
AT3G27380	SDH2-1; succinate dehydrogenase 2-1
AT1G76030	VAB1; ATPase, V1 complex, subunit B protein
AT4G38510	VAB2; ATPase, V1 complex, subunit B protein
AT1G78900	VHA-A; vacuolar ATP synthase subunit A
