# Supplementary material for: Proteome Dynamics of Persulfidation in Leaf Tissue under Light/Dark Conditions and Carbon Deprivation
Source: Antioxidants (Basel). 2023 Mar 23;12(4):789. doi: 10.3390/antiox12040789 (PMC10135009; doi:10.3390/antiox12040789)
Supplement: Supplementary file 1 [file antioxidants-12-00789-s001.zip › Table S3.pdf]

Table S3. Identified proteins in light clusters according to GO_BF and KEGG classifications.

AGI_ID	Gene name
Proteosome	
AT3G22110	20S proteasome alpha subunit C1 (PAC1)
AT1G53850	20S proteasome alpha subunit E1 (PAE1)
AT2G27020	20S proteasome alpha subunit G1 (PAG1)
AT3G51260	20S proteasome alpha subunit PAD1 (PAD1)
AT3G22630	20S proteasome beta subunit D1 (PBD1)
AT4G14800	20S proteasome beta subunit D2 (PBD2)
AT1G13060	20S proteasome beta subunit E1 (PBE1)
AT1G56450	20S proteasome beta subunit G1 (PBG1)
AT2G05840	20S proteasome subunit PAA2 (PAA2)
AT1G45000	AAA-type ATPase family protein
AT3G26340	N-terminal nucleophile aminohydrolases
AT4G31300	N-terminal nucleophile aminohydrolases
AT3G27430	N-terminal nucleophile aminohydrolases
AT3G60820	N-terminal nucleophile aminohydrolases
AT5G35590	proteasome alpha subunit A1 (PAA1)
AT5G66140	proteasome alpha subunit D2 (PAD2)
AT5G42790	proteasome alpha subunit F1 (PAF1)
AT1G21720	proteasome beta subunit C1 (PBC1)
AT3G53970	proteasome inhibitor-like protein
AT1G16470	proteasome subunit PAB1 (PAB1)
Glutathione metabolism	
AT1G64190	6-phosphogluconate dehydrogenase family protein
AT3G02360	6-phosphogluconate dehydrogenase family protein
AT5G41670	6-phosphogluconate dehydrogenase family protein
AT1G44790	ChaC-like family protein
AT2G24200	Cytosol aminopeptidase family protein (LAP1)
AT4G30920	Cytosol aminopeptidase family protein (LAP2)
AT5G02790	Glutathione S-transferase family protein (GSTL3)
AT5G14590	Isocitrate/isopropylmalate dehydrogenase family protein
AT1G63770	Peptidase M1 family protein
AT4G32320	ascorbate peroxidase 6(APX6)
AT1G75270	dehydroascorbate reductase 2 (DHAR2)
AT1G19570	dehydroascorbate reductase (DHAR1)
AT3G27300	glucose-6-phosphate dehydrogenase 5(G6PD5)
AT4G23100	glutamate-cysteine ligase (GSH1)
AT4G02520	glutathione S-transferase PHI 2 (GSTF2)
AT1G59700	glutathione S-transferase TAU 16 (GSTU16)
AT1G10360	glutathione S-transferase TAU 18 (GSTU18)
AT1G78380	glutathione S-transferase TAU 19 (GSTU19)
AT1G78340	glutathione S-transferase TAU 22 (GSTU22)
AT1G17170	glutathione S-transferase TAU 24 (GSTU24)
AT5G17220	glutathione S-transferase PHI 12 (GSTF12)
AT1G27130	glutathione S-transferase TAU 13 (GSTU13)
AT3G43800	glutathione S-transferase TAU 27 (GSTU27)
AT2G29470	glutathione S-transferase TAU 3 (GSTU3)
AT2G25080	glutathione peroxidase 1 (GPX1)
AT2G31570	glutathione peroxidase 2 (GPX2)
AT4G11600	glutathione peroxidase 6 (GPX6)
AT4G31870	glutathione peroxidase 7 (GPX7)
AT1G63460	glutathione peroxidase 8 (GPX8)
AT5G27380	glutathione synthetase 2 (GSH2)

AT3G55040	glutathione transferase lambda 2 (GSTL2)
AT1G70310	spermidine synthase 2 (SPDS2)
AT5G53120	spermidine synthase 3 (SPDS3)
AT1G23820	spermidine synthase 1 (SPDS1)
Nucleosome assembly	
AT2G28720	Histone superfamily protein
AT5G59910	Histone superfamily protein (HTB4)
AT3G45980	Histone superfamily protein (HTB9)
AT1G74560	NAP1-related protein 1 (NRP1)
AT1G18800	NAP1-related protein 2 (NRP2)
AT5G22880	histone B2 (HTB2)
AT2G19480	nucleosome assembly protein 1;2 (NAP1;2)
AT5G56950	nucleosome assembly protein 1;3 (NAP1;3)
AT4G26110	nucleosome assembly protein1;1 (NAP1;1)
AT1G06760	winged-helix DNA-binding transcription factor family protein
Glycolysis	
AT2G24270	aldehyde dehydrogenase 11A3 (ALDH11A3)
AT1G54100	aldehyde dehydrogenase 7B4(ALDH7B4)
AT5G03690	Aldolase superfamily protein (FBA4)
AT2G36460	Aldolase superfamily protein (FBA6)
AT2G01140	Aldolase superfamily protein (PDE345)
AT1G74030	Enolase 1 (ENO1)
AT2G36530	Enolase 2 (LOS2)
AT3G04120	glyceraldehyde-3-phosphate dehydrogenase C subunit 1 (GAPC1)
AT5G24760	GroES-like zinc-binding dehydrogenase family protein
AT5G61580	phosphofructokinase 4(PFK4)
AT1G12000	Phosphofructokinase family protein
AT1G20950	Phosphofructokinase family protein
AT1G76550	Phosphofructokinase family protein
AT5G51820	phosphoglucomutase (PGM)
AT1G70730	Phosphoglucomutase/phosphomannomutase family protein (PGM2)
AT1G23190	Phosphoglucomutase/phosphomannomutase family protein (PGM3)
At3g50520	Phosphoglycerate mutase family protein
AT1G59900	pyruvate dehydrogenase complex E1 alpha subunit (E1 ALPHA)
AT3G52990	Pyruvate kinase family protein
AT5G42740	Sugar isomerase (SIS) family protein
