# Supplementary figures and images for: Proteome Dynamics of Persulfidation in Leaf Tissue under Light/Dark Conditions and Carbon Deprivation
Source: Antioxidants (Basel). 2023 Mar 23;12(4):789. doi: 10.3390/antiox12040789 (PMC10135009; doi:10.3390/antiox12040789)

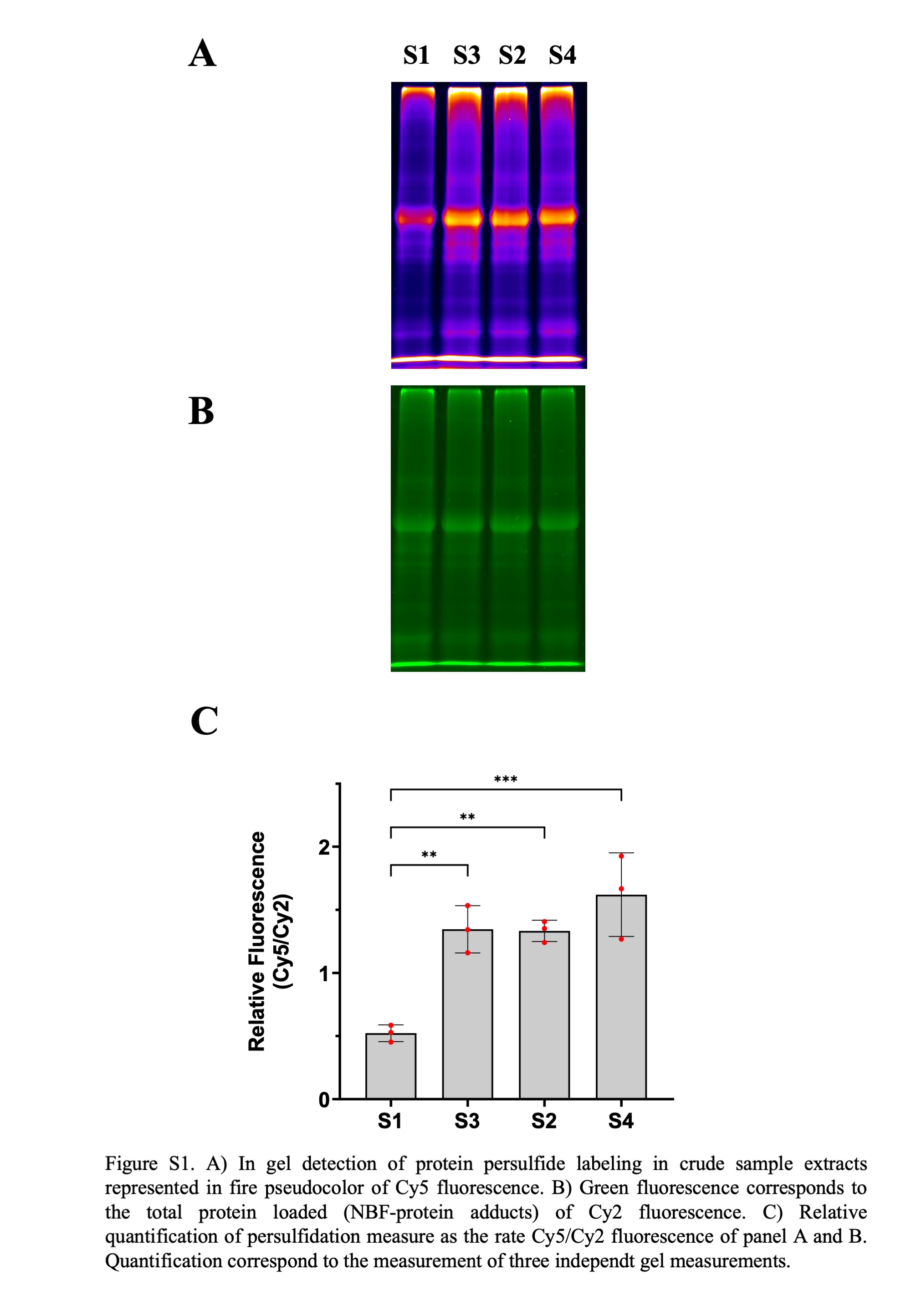

Supplement: Supplementary file 1 [file antioxidants-12-00789-s001.zip › Figure S1.jpg]

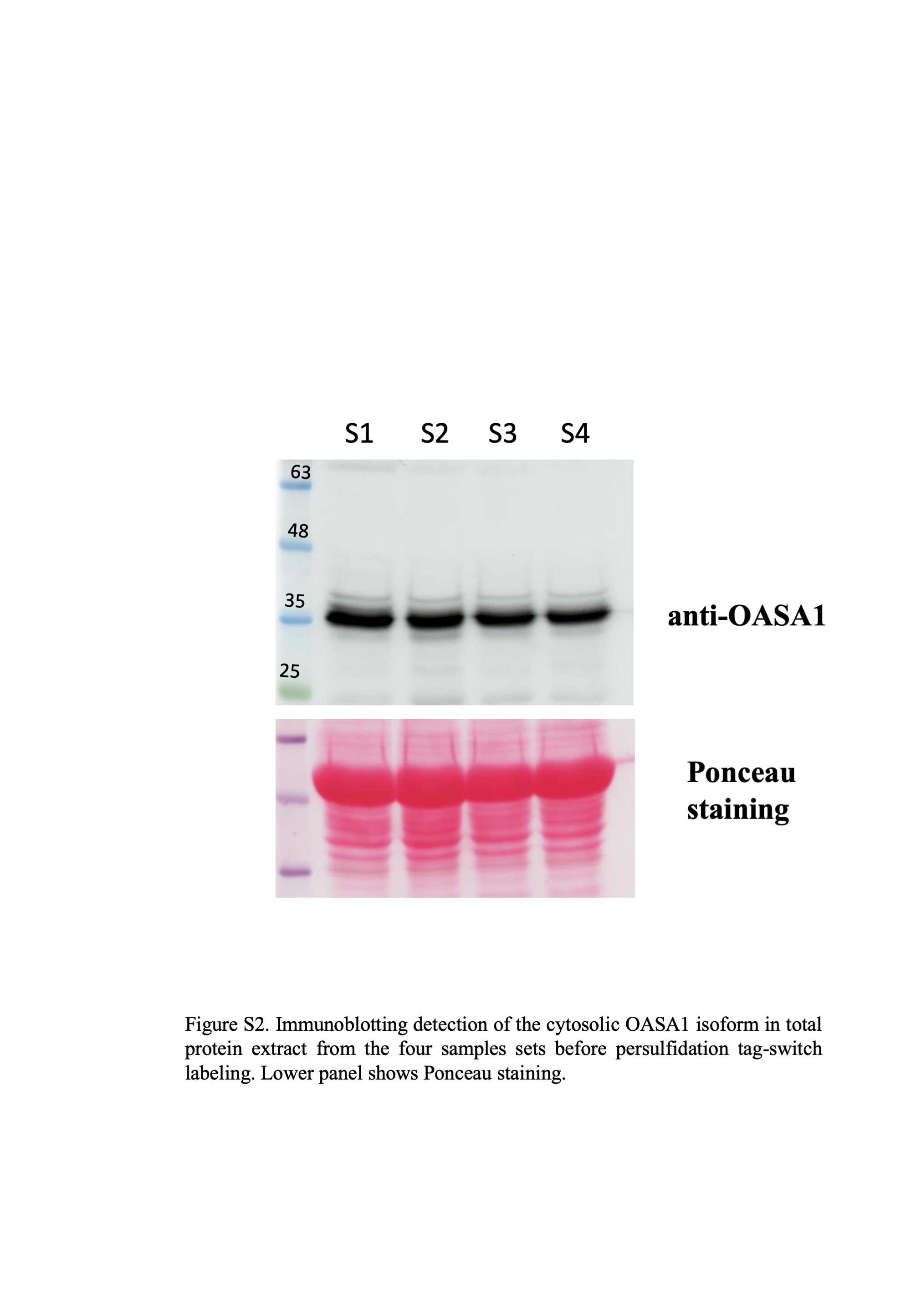

Supplement: Supplementary file 1 [file antioxidants-12-00789-s001.zip › Figure S2.jpg]

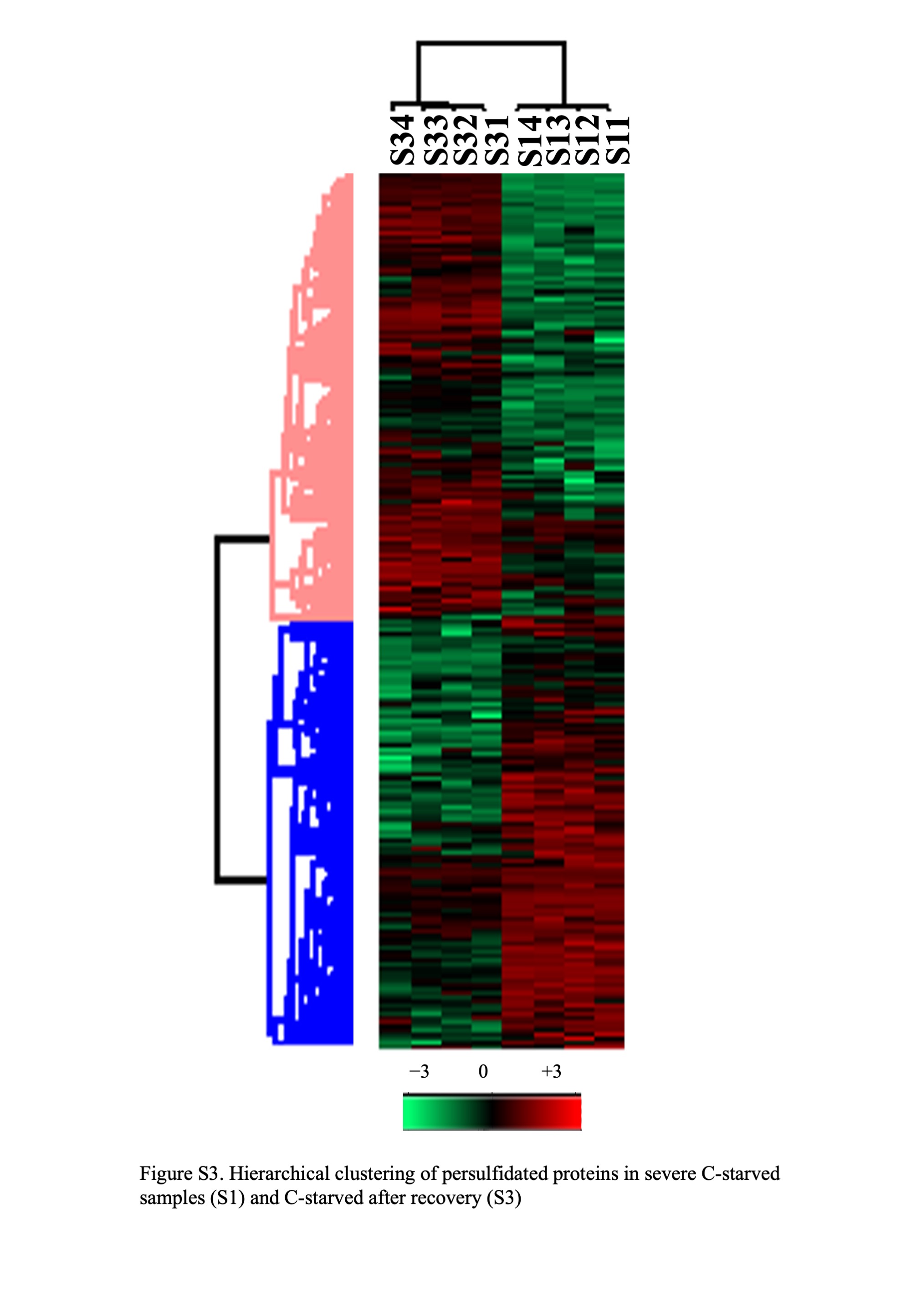

Supplement: Supplementary file 1 [file antioxidants-12-00789-s001.zip › Figure S3.jpg]
